# Intentional cystotomy in surgery for placenta percreta with bladder invasion: Not only for hysterectomy but also for uterus‐preserving surgery

**DOI:** 10.1111/aogs.14484

**Published:** 2022-11-30

**Authors:** Shigeki Matsubara

**Affiliations:** ^1^ Department of Obstetrics and Gynecology Jichi Medical University Tochigi Japan; ^2^ Department of Obstetrics and Gynecology Koga Red Cross Hospital Ibaraki Japan

Sir,

Placenta percreta with bladder invasion is the severest type of placenta accreta spectrum (PAS). In 2009, for the first time to our knowledge, we reported a detailed description of intentional cystotomy (“opening the bladder technique”) in cesarean hysterectomy for this disorder.^1^ At that time, this technique looked to attract less attention than we expected, possibly due to the relative rarity of this disorder.

More than one decade has passed. PAS incidence has increased. Recently, Feng et al.^2^ refocused this technique. Of note, they used this technique not for cesarean hysterectomy but for uterus‐preserving surgery (partial uterine wall resection and repair) with good outcomes. They referred to it as “sandwich excision”. Irrespective of the naming of opening the bladder or sandwich excision, I wish to share the significance of intentional cystotomy with the readers.

**FIGURE 1:**
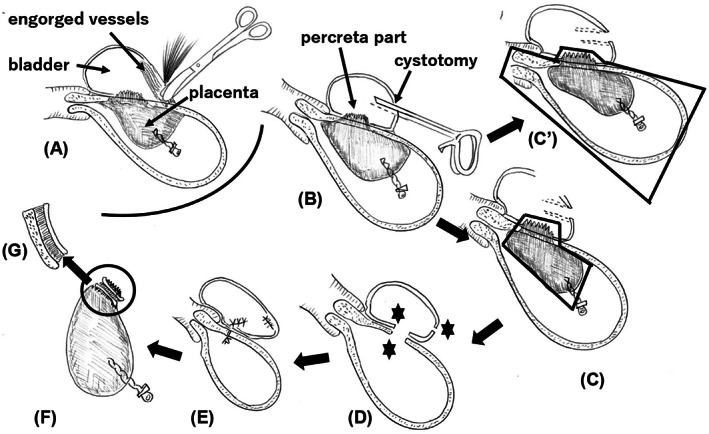
Schematic presentation of the opening the bladder technique or sandwich excision in patients with placenta percreta with bladder invasion. a: Engorged vessels are usually present at the vesicouterine plica and around the bladder top. Extensive bladder separation usually injures the engorged vessels, causing marked bleeding. b: With no, or at least less extensive, separation of the bladder, the bladder should be intentionally cut (cystotomy). An autosuture apparatus (Endo GIA, Covidien, Mansfield, MA, USA) is useful. c: In uterus‐preserving surgery, from this cystotomy window, the bladder wall and uterine wall should be resected with the placenta attached it. Usually, the peripheral parts of the placenta do not form increta or percreta and thus are removable. Black indicates the area that should be resected/removed. c': If hysterectomy is chosen, without uterine‐wall‐resection, hysterectomy should be performed with the resected bladder wall attached to the uterine wall (black). In either case (c or c'), the bladder can be incised in one or two sites depending on the situation of engorged vessels, bladder invasion, and the location of bladder‐top. For simplicity, the figure illustrates two bladder‐incisions. d: The cystotomy, bladder wall, and uterine wall should be repaired (star). As described, depending on the situation, cystotomy and the bladder wall resection can be done at the same (single) site, when this single site should be repaired. e: The bladder and uterine wall have been repaired. f: Removed specimen. From the upper: the resected bladder, placenta (percreta), and resected uterine wall with the placenta attached. g: A higher magnification of the area indicated by a circle in f. The bladder wall + placenta + uterine wall: this resembles a sandwich.

In anterior PAS, the placenta usually exists in the uterus‐bladder interface‐space, and vessels at the vesicouterine plica and around the bladder top are engorged: its extreme end is observed in placenta percreta with bladder invasion. In either hysterectomy or uterus‐preserving surgery, extensive bladder separation injures the engorged vessels, causing marked bleeding (Figure 1a).^3^, ^4^ Thus, without attempting to extensively separate the bladder, the bladder wall should be cut (intentional cystotomy; “opening the bladder”) (Figure 1b).^1^, ^4^ In hysterectomy, the invaded bladder wall should be cut without uterine‐wall‐resection. With the resected bladder‐wall attached to the uterine wall, hysterectomy should be performed, as previously described (Figure 1c').^1^, ^4^ In uterus‐preserving surgery, the part of the invaded bladder should be resected together with the uterine wall and placenta (Figure 1c).^2^ The bladder and uterus should be reconstructed (Figure 1d,e). Reconstruction of the intentionally incised bladder is easier than that of injured/damaged bladder. The resected specimen looks like a sandwich (Figure 1f,g). That is the reason why Feng et al.^2^ referred to this technique as “sandwich excision”.

In either case, the engorged vessels should not be, at least extensively, touched, which reduces the amount of bleeding. As previously described,^3^, ^4^ filling the bladder technique (the bladder being filled with 200–300 ml of water) may help us to recognize the topological relation of the bladder, lower uterine segment, and engorged vessels. When separation is needed, this enables us to perform bladder separation from the appropriate site, and to determine the appropriate cystotomy site, irrespective of hysterectomy or uterus‐preserving surgery.^3^, ^4^

I must describe two points. First, it has increasingly been acknowledged that “invasion” outside the uterus is biologically implausible. The condition of “percreta with/without bladder invasion” may be mainly due to uterine rupture or dehiscence and not due to the villous invasion.^5^ Second, careful ligation of the vessels and dissection of the space to create sufficient space to perform either a resection and repair or a hysterectomy is an acceptable alternative. I believe that intentional cystotomy can be an option but this is based on my personal impression. I do not claim that intentional cystotomy is the only technique. I only wish to state that accumulation of surgical experience will improve the PAS surgery.
